# Comparison of Interscalene Brachial Plexus, Anterior Suprascapular Nerve, and Costoclavicular Brachial Plexus Blocks in Arthroscopic Shoulder Surgery: A Prospective Observational Study

**DOI:** 10.3390/jcm15020421

**Published:** 2026-01-06

**Authors:** Burak Taha Sarıoğlan, Yeliz Kılıç, İrem Eraslan Sarıoğlan, Mehmet Sacit Güleç

**Affiliations:** 1 Department of Anesthesiology and Reanimation, Gaziantep City Hospital, Gaziantep 27470, Türkiye; 2 Department of Anesthesiology and Reanimation, Faculty of Medicine, Osmangazi University, Büyükdere Mh, Odunpazarı, Eskişehir 26040, Türkiye

**Keywords:** anterior suprascapular nerve block, costoclavicular brachial plexus block, diaphragm paralysis, interscalene brachial plexus block, postoperative pain

## Abstract

**Background:** Interscalene brachial plexus block (ISB) remains the gold standard anesthesia method in shoulder surgery. However, risk of diaphragm paralysis is a major concern among anesthesiologists. Recent studies on anterior suprascapular nerve block (ASB) and costoclavicular brachial plexus block (CCB) have given promising results for preventing diaphragm paralysis and providing sufficient analgesia. **Methods:** Forty-six patients who underwent arthroscopic shoulder surgery under one of three regional anesthesia techniques, including ISB (*n* = 15), ASB (*n* = 15), and CCB (*n* = 16), were included in the study. Diaphragmatic excursion was measured by ultrasonography 30 min after the block. Postoperative pain was assessed with a numerical rating scale. The groups were compared in terms of diaphragm paralysis and postoperative pain status. **Results:** The groups were similar in basic patient and surgical characteristics as well as motor and sensory block scores. There was no difference in analgesic use between the groups. Diaphragm measurements in the ISB group were found to be significantly lower compared to the ASB and CCB groups (*p* < 0.001). In addition, diaphragm measurements in the ASB group were found to be lower than in the CCB group (*p* = 0.036). When comparing diaphragm measurements between the initial and 30th min of block, significant decreases were observed in the ISB and ASB groups (*p* < 0.001), whereas no difference was found in the CCB group. **Conclusions:** Postoperative pain scores and analgesic use were similar between the three blocks. In terms of diaphragm paralysis, the best blocks appeared to be CCB followed by ASB. CCB and ASB can be considered as safe and effective alternative blocks in arthroscopic shoulder surgery, particularly for patients without serious obstructive or restrictive pulmonary disease.

## 1. Introduction

Although arthroscopic techniques are associated with good cosmetic results, shorter hospitalization, and increased patient satisfaction, postoperative pain remains a significant problem in a portion of patients undergoing shoulder surgery [1,2,3]. Pain not only impairs the patient’s comfort but also affects postoperative functional results by hindering early rehabilitation. Intravenous (IV) opioids are among the most commonly used analgesia tools for postoperative pain control. However, undesirable side effects such as respiratory depression, prolonged sedation, constipation, allergic reactions, nausea, and vomiting limit its wide use [4]. In this context, different regional anesthesia techniques are being introduced into the routine practice for minimizing those problems and providing better analgesia. Recent advances in ultrasonography (US) techniques, such as the use of high-frequency probes in identifying peripheral nerves and providing clearer imaging of superficial tissues, have allowed safer, faster, and more comfortable blocks [5].

Interscalene brachial plexus block (ISB) is considered as a gold standard anesthesia method in shoulder surgery; however, risk of diaphragm paralysis due to phrenic nerve injury is its leading disadvantage [6,7]. Changes in diaphragm functions can be measured and monitored by sonographic examination of the respiratory system, allowing the decision of correct block type on a patient-by-patient basis [8]. Recent studies on anterior suprascapular nerve block (ASB) and costoclavicular brachial plexus block (CCB) have given promising results for providing sufficient analgesia and minimizing diaphragm paralysis [9,10]. However, this study is the first in the current literature to include a simultaneous comparison of three blocks, ISB, ASB, and CCB, under uniform conditions, such as using US and nerve stimulators.

Based on these considerations, the present study aims to compare the effects of the ISB, ASB, and CCB techniques on postoperative pain and diaphragm functions in patients undergoing arthroscopic shoulder surgery.

## 2. Materials and Methods

### 2.1. Study Design

This prospective observational study was conducted at the Department of Anesthesiology and Reanimation, Eskişehir Osmangazi University, after receiving approval from the Institutional Clinical Research Ethics Committee (date: 9 February 2023, No.: 47). The patients were informed in detail about the stages of the study, and both verbal and written informed consents were obtained from all.

Forty-six patients who underwent arthroscopic shoulder surgery under one of three regional anesthesia techniques, including ISB (*n* = 15), ASB (*n* = 15), and CCB (*n* = 16), were included in the study. The primary outcome was the differences in postoperative pain scores and analgesic use between the three regional blocks. The secondary outcomes were the differences in diaphragm functions, motor and sensory block scores, postoperative nausea and vomiting, and patient and surgeon satisfaction between the blocks.

Inclusion criteria were being above 18 years old, having an American Society of Anesthesiologists (ASA) status of 1–3 and body mass index (BMI) between 20 and 40. The patients with neuropathic disorder, coagulopathy (preoperative platelet < 100 μL, Inr > 1.5, or prothrombin time > 50 s), obstructive or restrictive lung disease, renal/hepatic failure, allergy to local anesthetics, history of previous neck surgery, opioid use due to chronic pain syndrome, and pregnancy were excluded from the study.

Regional blocks were performed by an anesthesiologist (Burak Taha Sarıoğlan, MD), whereas assessments of diaphragm excursion, motor and sensory functions, and postoperative pain were performed by another anesthesiologist who was blinded to the regional block type performed.

### 2.2. Assessment of Diaphragm Excursion Before Regional Block

The patients were placed on the operating table in a supine position. Standard monitoring included heart rate (HR), peripheral oxygen saturation (SpO_2_), and blood pressure (BP). Nasal oxygen (2 L/min) was administered, and IV access was established with a 20-gauge branula. Patients were premedicated with IV midazolam (2 mg). Diaphragm excursion on the shoulder side to be operated was assessed with a 2–5 MHz convex US probe (Philips Affiniti 50, Philips Medical Systems, Seattle, WA, USA) through subcostal acoustic windows between the anterior axillary midclavicular line adjacent to the liver or spleen. Inspiratory and expiratory craniocaudal displacements were measured as a bright line (hyperechoic waves) during normal breathing, by switching from B mode to M mode. Excursion values obtained from ultrasound measurements were interpreted based on the cut-off values specified in the study by Boussuges et al. [11] (Table 1). Hemidiaphragmatic paralysis was defined as the absence of diaphragmatic movement during normal (quiet) breathing, the absence of cranial diaphragmatic movement when the patient forcefully inhales, or (paradoxically) the absence of cranial diaphragmatic movement. At least 25% loss in diaphragmatic movement was considered significant.

### 2.3. Basic Data Regarding Regional Block

After the sonographic assessment of diaphragm excursion, the patients were divided into three groups: ISB, ASB, or CCB. A high-frequency 5–13 MHz linear US probe, a 50 mm long 22-G short-beveled regional block needle, and a nerve stimulator (Vygon Ltd., Swindon, UK) were used in all blocks. The nerve stimulator was set to provide a 1.5–2.5 mA current at a frequency of 1 Hz and a pulse duration of 0.1 ms. The needle was directed downward and inward through the skin. Local anesthetic was administered when stimulation was received at 1 mA and when stimulation was lost at 0.3 mA.

### 2.4. Interscalene Brachial Plexus Block (ISB) (Figure 1A)

In the supine position, with the arm adducted, the probe was placed transversely in the supraclavicular fossa just proximal to the midpoint of the clavicle. The brachial plexus was identified as a bright echogenic structure, posterolateral to the subclavian artery (bunch of grapes sign). Then, the nerves were held in the center of the screen, and the probe was moved in a cephalic direction up to the interscalene groove until a “string of pearls” image was obtained of the nerve roots located between the anterior and middle scalene muscles. After optimizing the image of the nerve roots as hypoechoic round or oval structures, the needle was placed posterolateral to the probe and moved in the plane. The needle was then located between the middle scalene muscle and the brachial plexus roots, and 15 mL of 0.25% bupivacaine was slowly injected into the area between the C5-6 roots.

**Figure 1:**
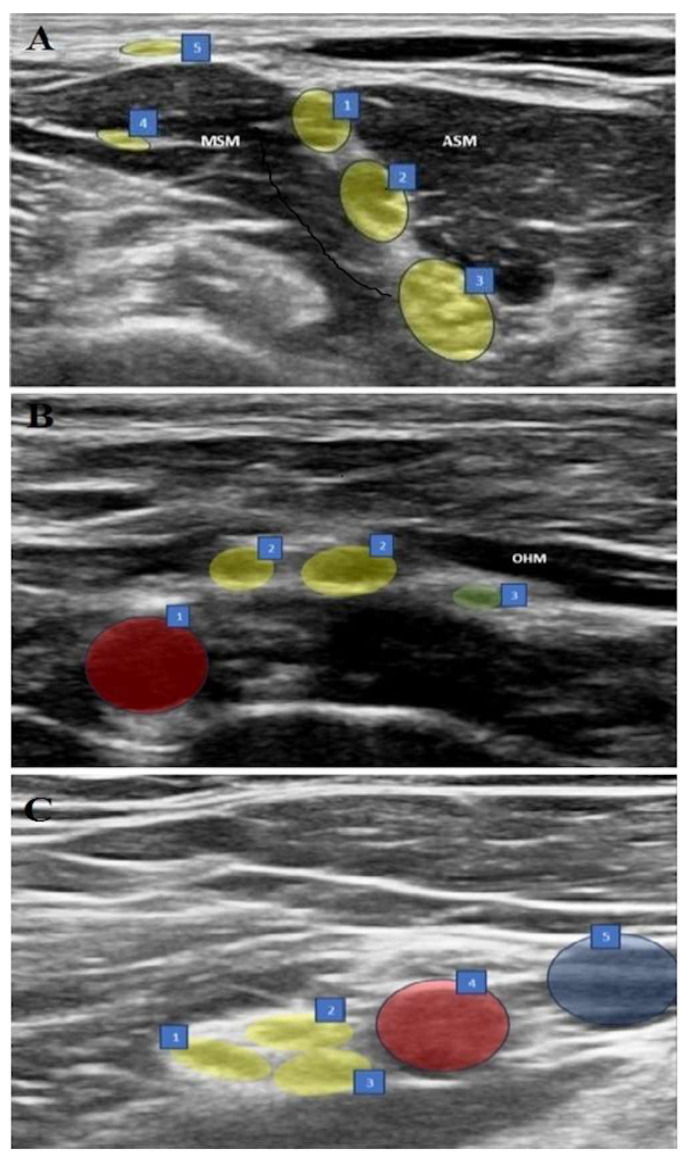
(**A**–**C**) Sonographic views of three blocks. (**A**) ISB: 1. C5 nerve root, 2. C6 nerve root, 3. C7 nerve root, 4. Long thoracic nerve, 5. Supraclavicular nerves, ASM: Anterior scalene muscle, MSM: Middle scalene muscle. (**B**) ASB: 1. Subclavian artery, 2. Upper trunk, 3. Suprascapular nerve, OHM: omohyoid muscle. (**C**) CCB: 1. Posterior cord, 2. Lateral cord, 3. Medial cord, 4. Axillary artery, 5. Axillary vein.

### 2.5. Anterior Suprascapular Nerve Block (ASB) (Figure 1B)

In the supine position, with the arm adducted, the probe was placed in the supraclavicular fossa to identify the brachial plexus. The plexus was then followed until the suprascapular nerve branching from the upper trunk was seen. The needle was advanced from posterolateral to anteromedial, and the most lateral transverse image of the nerve was obtained with the in-plane technique. The nerve was entered between the superficial cervical fascia and prevertebral fascia under the omohyoid muscle from the lateral side, and 5 mL of 0.25% bupivacaine was injected following hydrodissection with DW5 and negative aspiration.

### 2.6. Costoclavicular Brachial Plexus Block (CCB) (Figure 1C)

The ipsilateral arm was abducted at 90 degrees with the palm facing the ceiling, and the ultrasound transducer was positioned parallel to the mid-clavicle. The long axis of the transducer was tilted slightly cephalad, and the ultrasound beam was directed into the costoclavicular space. The ultrasound image was optimized so that all three cords of the brachial plexus were seen together lateral to the axillary artery, anteriorly between the clavicular head of the pectoralis major and the subclavius muscle and posteriorly between the serratus anterior muscle overlying the second rib. The needle was then advanced laterally and medially in the plane. With the needle tip in its intended position, a small volume (0.5–1 mL) of DW5 was injected before each injection to ensure that the needle tip was within the hyperechoic connective tissue matrix underlying the brachial plexus sheath and between the cords of the brachial plexus and not intraneurally. After verification, the local anesthetic was injected into multiple sites in 4–5 mL aliquots by redirecting the needle, for a total of 20 mL of 0.25% bupivacaine, to homogeneously distribute the local anesthetic.

### 2.7. Motor and Sensory Assessments of the Regional Blocks

Thirty minutes after the block, ipsilateral hemidiaphragmatic excursion was measured and recorded at normal depth of breath. Motor and sensory functions of the shoulder were evaluated by a blind observer at 5 min intervals for 30 min after the block. Motor function was evaluated with shoulder abduction (axillary and suprascapular nerves) and lateral rotation of the humerus against manual resistance (suprascapular nerve) while the arm was adducted and the elbow was flexed to 90°, with a score between 0 and 3 (0 = no motor block, 1 = no shoulder abduction, 2 = no shoulder abduction or elbow flexion, 3 = complete motor block). At the same time, sensory function was evaluated with the “pinprick test” (2 = feeling touch, 1 = reduced sense of touch, 0 = no sensation) on the skin over the clavicle (supraclavicular nerves) and the lateral surface of the deltoid (axillary nerve), and when the response to the pinprick test disappeared, it was considered ready for general anesthesia.

### 2.8. Induction and Maintenance of Anesthesia

In each group, induction was performed with IV propofol (1.5–2.5 mg/kg), IV fentanyl (1.5–3 μg/kg), and IV rocuronium (0.6 mg/kg). Anesthesia was maintained with sevoflurane (1–1.3 MAC) in a 50%/50% O_2_/air mixture and remifentanil infusion (0.01–0.1 μg/kg/min). Mechanical ventilator settings were adjusted to a tidal volume of 6–8 mL/kg and end-tidal CO_2_ of 30–35 mmHg. Arthroscopic shoulder surgery was performed on the patients in the beach bed position by the same surgical team. The patients’ hemodynamic data were recorded throughout the operation.

### 2.9. Postoperative Assessments

All patients were followed-up at the 1st, 2nd, 6th, 12th, and 24th hours postoperatively. The patients’ alertness was recorded at the postoperative 1st, 2nd, 6th, 12th, and 24th hours with the Ramsay sedation scale. Scores of 1-2-3 on this scale indicate that the patient is awake, and scores of 4-5-6 indicate that the patient is under sedation.

Postoperative pain level was assessed with an 11-point numerical rating scale (NRS) ranging from 0 to 10. As part of multimodal pain management, all patients were given routine 1 g IV paracetamol (3 times daily). If NRS was >4, IV dexketoprofen (50 mg/2 mL) was administrated. If there was insufficient response to dexketoprofen, IV tramadol (1 mg/kg) was added.

Patients’ motor and sensory complaints in the ipsilateral upper extremity, side effects, and complications were recorded at the 1st, 2nd, 6th, 12th, and 24th hours. Postoperative nausea and vomiting were assessed with the Likert scale (Table 2). Patient and surgeon satisfaction were assessed with a 5-point scale at the 24th hour postoperatively (5—very satisfied, 4—satisfied, 3—undecided, 2—dissatisfied, 1—very dissatisfied).

### 2.10. Statistical Analysis

The sample size and power analysis of the study were performed using G power (version 3.1.9.4, using ANOVA test), based on the values obtained from the hemidiaphragm paralysis measurements in the study by Auyong et al. comparing the effects of interscalene, supraclavicular, and anterior suprascapular blocks on postoperative pain and diaphragm functions in shoulder surgery patients [12]. It was calculated that 80% power and 5% type I error could be achieved if at least 30 patients were included in the study (at least 10 patients for each group). The groups were determined as consisting of 15 people, considering possible follow-up losses. Data were analyzed using the SPSS 26 (SPSS Inc., Chicago, IL, USA) package program. Continuous and categorical variables were expressed as mean ± standard deviation (minimum–maximum) and numbers (percentages), respectively. Variables that conformed to normal distribution were examined with parametric tests, and variables that did not conform to normal distribution were examined with non-parametric tests. When parametric test assumptions were met, one-way analysis of variance (ANOVA), which examines the differences between three groups, was used in comparing independent group differences; when parametric test assumptions were not met, the Kruskal–Wallis test, which is a non-parametric test that examines the differences between three groups, was used in comparing independent group differences. Sphericity was analyzed using Mauchly’s Sphericity Test, and corrections were applied using Greenhouse–Geisser or Huynh–Feldt tests when the *p*-value was less than 0.05. A *p*-value less than 0.05 was considered statistically significant.

## 3. Results

The data of 46 patients with a mean age of 51.4 years old were analyzed. There were three groups according to the regional anesthesia technique: ISB (*n* = 15), ASB (*n* = 15), and CCB (*n* = 16). All groups were similar in basic characteristics (Table 3).

In terms of motor and sensorial block examinations performed at the 30th min, no significant differences were found between the groups (*p* = 0.345 and *p* = 0.230, respectively). Complete motor and sensorial blocks were observed in all patients.

There was no significant difference between the groups in the use of 0–6th, 6–12th and 6–12th hour non-steroidal anti-inflammatory drugs (NSAIDs) and rescue analgesia (tramadol) (*p* > 0.05) (Table 4).

The mean initial normal breath diaphragm measurements were 1.97 ± 0.22 cm in the ISB group, 1.81 ± 0.15 cm in the ASB group, and 1.75 ± 0.17 cm in the CCB group. Initial values were significantly higher in the ISB group compared to the CCB group (*p* = 0.006). No significant difference was observed in the pairwise comparisons of other initial values (*p* > 0.05). At 30 min after the block, diaphragm measurements in the ISB group were found to be significantly lower compared to the ASB and CCB groups (*p* < 0.001). In addition, diaphragm measurements in the ASB group were found to be significantly lower compared to the CCB group (*p* = 0.036). In each group, changes in diaphragm measurements between the beginning and the 30th min were compared; significant decreases were observed in the ISB and ASB groups (*p* < 0.001), while no difference was found in the CCB group (*p* > 0.05) (Table 5).

No significant difference was observed in Ramsay sedation (RS) scores among the groups (*p* > 0.05). All groups were similar in terms of pre-block NRS scores (*p* > 0.05). At 1 and 2 h, the NRS scores of the ISB group were significantly lower than those of the ASB and CCB groups (*p* < 0.001), while there was no significant difference between the ASB and CCB groups (*p* > 0.05). No significant difference was found in the pairwise comparisons of the three groups in terms of NRS scores at 6 h (*p* > 0.05). NRS scores at 12 and 24 h were lower in the ASB group than in the ISB group (*p* < 0.05). At 12 and 24 h, no significant differences were found between CCB and ISB and between CCB and ASB (*p* > 0.05).

No differences were found between the groups in terms of postoperative nausea (*p* = 0.764) and vomiting (*p* = 0.852) scores. There were also no differences between the groups in terms of patient satisfaction (*p* = 0.611) and surgeon satisfaction (*p* = 0.450).

One patient in the CCB and ISB groups had paresthesia, and one patient in the ISB group developed Horner syndrome. No significant difference was found between the groups in terms of complications (*p* = 0.607).

## 4. Discussion

In the present study, three different brachial plexus blockade techniques, ISB, ASB, and CCB, were compared in terms of diaphragm functions and analgesia in patients undergoing shoulder arthroscopy. The results showed that three techniques provided effective shoulder analgesia but had different effects on diaphragm functions.

Shoulder innervation is provided by the distal branches of the C5-6 roots, including suprascapular, axillary, subscapular, and lateral pectoral nerves [13]. Recently, the suprascapular nerve has been demonstrated not to be the dominant nerve of shoulder innervation as previously described [14]. In clinical studies, posterior suprascapular blockade, which blocks only the innervation of the posterior upper quadrant of the shoulder, has been shown to have limited advantage over placebos and has no clinically significant effect on pain scores and opioid consumption [15]. Currently, the subscapular and axillary nerves are known to contribute to the innervations of the anterior upper quadrant of the shoulder joint and the lower half of the joint, respectively [14]. These findings have led researchers to combine posterior suprascapular blockade with additional blocks such as supraclavicular [16] and infraclavicular [17] techniques or to find new block locations. ASB, which exposes the suprascapular nerve more proximally and anteriorly, has been presented as a reliable alternative to the shortcomings of the posterior approach to the suprascapular nerve [18].

In the present study, there was a significant difference in favor of ISB in the first two hours; however, this difference disappeared at 6 h, and it was found that the ASB group showed significantly better analgesia at 12–24 h. These results supported the previous finding that the upper trunk is consistently involved in ASB.

Contrary to previous belief, the fact that the posterior rather than anterior portion of the upper trunk is located close to the suprascapular nerve may explain the analgesic efficacy of the ASB [19]. The posterior portion of the upper trunk and thus the axillary and subscapular nerves innervating the shoulder can be blocked by directing a low volume of local anesthetic to the proximal origin of the suprascapular nerve [12]^.^

The fact that the distance between the phrenic nerve and the C5 root (and also the suprascapular nerve) enlarges towards the root of the neck is another advantage of ASB, resulting in minimization of the effect of the local anesthetic on the phrenic nerve. In parallel, the diaphragm measurements at 30th min were lower in the ISB group compared to the ASB group. The incidence of hemidiaphragmatic paresis after ISB increases to 100% with local anesthetic volumes of 20 mL or more [20]. This can be reduced by up to 45% by reducing the local anesthetic volume to 5–10 mL but is associated with a clinically significant decrease in the duration and efficacy of perioperative analgesia and carries a risk of block failure [20,21,22]. Therefore, in our study, a standard dose of 15 mL of 0.25% bupivacaine was used for ISB. The local anesthetic concentration was determined as 0.25% in order to reduce the differences in total drug doses administered between the groups. On the other hand, the differences in local anesthetic volumes between the blocks are related to the application sites of these drugs; in other words, the three block types differ technically and in their application areas. Local anesthetic doses were determined in accordance with routine practice, at optimal volumes and concentrations that minimize phrenic nerve involvement while providing sufficient analgesia. In our study, 5 mL of 0.25% bupivacaine was used in the ASB group, as in previous studies [10]. The diaphragm function at the 30th min was better in the ASB group than in the ISB group. However, the significant decrease in the 30th min value compared to the initial level was a remarkable result. In three studies where the upper trunk block was evaluated in terms of preserving diaphragmatic and respiratory functions, the rates of hemidiaphragmatic paralysis were reported to be significantly different (4.8%, 5.3%, and 54.3%) using the same volume (15 mL) and the same diagnostic tool (ultrasound evaluation of diaphragm excursion) [23,24,25]. In a recent cadaver study, it was reported that ASB performed with a mixture containing 5 mL of methylene blue stained the superior trunk 100% and caused 20% retrograde staining in the phrenic nerve [26]. However, it should be kept in mind that the results of cadaver studies may not always be consistent with clinical findings. Different volumes and injection techniques for ASB should be evaluated in future studies for optimization of diaphragm function.

Injury to the dorsal scapular and long thoracic nerves is another concern in ISB, because these nerves pass through the middle scalene muscle. In ASB, the needle does not pass through this muscle but courses between the superficial cervical and prevertebral fascias under the omohyoid muscle. Despite these advantages, the anatomical difficulties in ASB, such as the proximity to vascular structures including the suprascapular and transverse cervical arteries and the inability to see the suprascapular nerve branching off from the upper trunk, should not be forgotten.

CCB is a relatively new infraclavicular block technique that has become widespread due to its rapid and reliable blockade [27]. Since sensory innervation from the shoulder joint and adjacent structures is provided by the lateral cord (lateral pectoral nerve), posterior cord (axillary and subscapular nerves), and upper trunk (suprascapular nerve), a block approach that extends too far distally increases the risk of incomplete blockage of one of these neural structures (especially the suprascapular nerve) [7]. In the costoclavicular space, the three cords come together, maintaining a consistent relationship between them, and form the brachial plexus. Furthermore, this area is located more superficially than the area targeted by the classic infraclavicular approach. The advantages that make CCB popular are that it can reliably block the lateral and posterior cords, the supraclavicular brachial plexus, and suprascapular nerve via the retrograde channel and that the needle entry site is located far enough from the phrenic nerve and brachial plexus to prevent the risk of hemidiaphragmatic paralysis [28]. In a recent study that examined CCB as an alternative to the ISB for shoulder surgery, focusing on minimizing involvement of the phrenic nerve, the authors found that local anesthetic deposition in the costoclavicular space can reliably anesthetize the brachial plexus cords, achieving a high rate of suprascapular nerve blockade while sparing the diaphragm. The authors also noticed that the effectiveness of anesthesia and analgesia provided by CCB depends on use of the appropriate local anesthetic volume and concentration [29]. In our study, the volume was selected as 20 mL for blockade of the lateral and posterior cords (not the medial cord), as suggested by Karmakar et al. [30], and the concentration was set at 0.25% to reduce the difference between the total amounts of local anesthetic. It should be noted here that further studies are needed to determine the optimal (adequate analgesia and diaphragm-protecting) local anesthetic volume and concentration in CCB.

In terms of analgesia, a significant difference was found in favor of ISB at the 2nd hour. However, no difference was found between the analgesic efficacies of ISB and CCB at the 6th, 12th, and 24th hours. Also, no significant difference was observed between ASB and CCB in terms of pain scores at any of the 24 h follow-ups. Our results clearly showed the stable and sufficient analgesic efficacy of CCB. During CCB, the lateral pectoral, subscapular, and axillary nerves are completely blocked by lateral and posterior cord blockade [31]. However, the suprascapular nerve can only be blocked by the supraclavicular spread of local anesthetic. Several anatomical structures that may limit this spread have been defined, such as the presence of more than one intraplexus septum in the supraclavicular fossa [7]. In addition, the presences of a paraneural sheath and an intraplexus septum between the three cords in the costoclavicular area have been reported both in cadavers and living organisms [31,32]. These are believed to reduce the spread of the injection to the surrounding cords and proximal to the brachial plexus.

In our study, the diaphragm measurements at the 30th min after blockade were found to be significantly lower in the ISB group compared to the CCB group. Interestingly, the diaphragm measurements at the 30th min after blockade in the ASB group were also significantly lower than in the CCB group. As known, moving from the supraclavicular fossa to the infraclavicular fossa during shoulder analgesia further reduces the risk of hemidiaphragmatic paralysis [28,33]. The paraneural sheath and fascial compartments surrounding the costoclavicular space cords play critical roles in the spread of local anesthetic. The connective tissue and the three-cord organization create a potential retrograde channel, forcing the local anesthetic to spread around the cords and in a cephalic direction. On the other hand, the increase in the distance between the costoclavicular space and the interscalene groove with local anesthetic injection acts as a physical barrier that prevents the spread of local anesthetic to the phrenic nerve, stellate ganglion, and recurrent laryngeal nerve [34]. In our study, unlike the other groups, no significant change was detected between the beginning and the 30th min of diaphragm measurements in the CCB group. This proves the protective role of CCB against hemidiaphragmatic paralysis in the local anesthetic volumes used in our study, similar to previous studies [10,34].

There were several limitations to our study. First, all blocks were performed by a single anesthesiologist in a single center, which facilitated standardization but limited the generalizability of the results. Blocks were performed for analgesia in patients undergoing general anesthesia. Further studies are needed to determine the anesthetic adequacy of the ASB and CCB groups. In the study, blocks were performed only in patients undergoing shoulder arthroscopy, and the results may not be generalizable to other surgeries such as shoulder arthroplasty. All blocks were performed with a single injection technique, and the side effects that may develop due to catheter-based blocks and local anesthetic accumulation are the subject of other new studies. Since patient-controlled analgesia was not used in the postoperative period, the postoperative opioid consumption of the patients could not be measured. The heterogeneity in volumes and total doses of bupivacaine between the groups represents an intrinsic limitation that may have influenced both the duration of analgesia and the incidence of respiratory complications. Finally, only diaphragm excursion values were measured, and detailed respiratory function tests were not performed. Although no serious side effects were observed, it is important to be cautious in generalizing the results of the study, especially to patients with serious respiratory comorbidities.

## 5. Conclusions

Although the NRS score at the postoperative 2nd hour was significantly lower in the ISB group than in the ASB and CCB groups, subsequent measurements showed that adequate analgesia was provided in all groups. In terms of diaphragm paralysis, the best block appeared to be CCB. Similarly, ASB was found to be associated with a reduced risk of diaphragm paralysis. All patients in the ISB group developed diaphragm paralysis. These results showed that CCB and ASB can be considered as safe and effective alternative blocks in arthroscopic shoulder surgery, particularly for patients without serious obstructive or restrictive pulmonary disease.

## Figures and Tables

**Table 1:** Normal (cut-off) excursion values for different respiratory maneuvers according to gender [11].

	RIGHT		LEFT	
	Men	Women	Men	Women
Quiet breathing	1.8 ± 0.3	1.6 ± 0.3	1.8 ± 0.4	1.6 ± 0.4
Voluntary sniffing	2.9 ± 0.6	2.6 ± 0.5	3.1 ± 0.6	2.7 ± 0.5
Deep breathing	7.0 ± 0.6	5.7 ± 1.0	7.5 ± 0.9	6.4 ± 1.0

All values are in centimeters (cm) and presented as mean ± SD.

**Table 2:** Postoperative nausea and vomiting (Likert scale).

Nausea	Vomiting
0—None	0—None
1—Does not prevent eating	1—Once in 24 h
2—Significantly reduces oral intake	2—2–5 times in 24 h
3—Requires IV fluid administration	3—≥6 times in 24 h or IV fluid requirement
	4—Requires hospitalization

**Table 3:** Comparison of basic characteristics between the groups.

	ISB (*n* = 15)	ASB (*n* = 15)	CCB (*n* = 16)	*p*
Age (mean, y)	46.7 ± 10.6	53.6 ± 10.1	52.7 ± 14.8	0.167
Gender (F/M)	5/10	6/9	7/9	0.835
BMI (kg/m^2^)	27.2 ± 3	29.5 ± 5.7	26.6 ± 4.3	0.188
Lateralization (R/L)	9/6	9/6	9/7	0.970
ASA status				0.279
ASA 1	7	4	2	
ASA 2	8	10	13	
ASA 3	0	1	1	

Age and BMI are presented as mean ± SD; other variables are presented as number. y: Year, F: Female, M: Male, BMI: Body Mass Index, R: Right, L: Left, ASA: American Society of Anesthesiologists.

**Table 4:** Comparison of additional analgesic use between the groups.

	ISB (*n* = 15)	ASB (*n* = 15)	CCB (*n* = 15)	*p*
0–6th hour NSAID	5	6	3	0.441
6–12th hour NSAID	6	2	5	0.304
12–24th hour NSAID	3	2	1	0.497
Rescue analgesia (tramadol)	2	1	2	0.814

All variables are presented as numbers. NSAIDs: Non-steroidal anti-inflammatory drugs.

**Table 5:** Comparison of the initial and 30th min diaphragm measurements in each group (during normal breathing).

	Initial	30th min of Block	*p*
ISB	1.97 ± 0.22 cm	0.2 ± 0.1 cm	**<0.001**
ASB	1.81 ± 0.15 cm	1.36 ± 0.5 cm	**<0.001**
CCB	1.75 ± 0.17 cm	1.67 ± 0.2 cm	0.312

All variables are presented as mean ± SD. cm: centimeter.

## Data Availability

Datasets analyzed during the current study are not publicly available due to patient privacy limitations but are available from the corresponding author on reasonable request.

## Abbreviations

The following abbreviations are used in this manuscript:

IV	Intravenous
ISB	Interscalene brachial plexus block
ASB	Anterior suprascapular nerve block
CCB	Costoclavicular brachial plexus block
US	Ultrasonography
ASA	American Society of Anesthesiologists
BMI	Body mass index
HR	Heart rate
SpO_2_	Peripheral oxygen saturation
BP	Blood pressure
NRS	Numerical rating scale
